# Caracterización molecular de la nueva entidad clínica relacionada con la hiperplasia suprarrenal congénita, síndrome CAH-X en población española

**DOI:** 10.1515/almed-2023-0050

**Published:** 2023-07-04

**Authors:** Laura Martínez Figueras, Rafael Muñoz Pacheco, Dolores García González, María Arriba Domènech, Begoña Ezquieta Zubicaray

**Affiliations:** Laboratorio de Diagnóstico Molecular, Servicio de Bioquímica Clínica, Hospital Materno Infantil Gregorio Marañón, Instituto de Investigación Sanitaria Gregorio Marañón, Madrid, España; Laboratorio de Diagnóstico Molecular, Servicio de Bioquímica Clínica, Hospital General Universitario Gregorio Marañón, Madrid, España

**Keywords:** *CYP21A2*, Ehlers-Danlos hipermóvil, hiperplasia suprarrenal congénita, síndrome CAH-X, tenascina, *TNXB*

## Abstract

**Objetivos:**

La recombinación entre *CYP21A2*-*TNXB* y sus respectivos pseudogenes (*CYP21A1P*-*TNXA*) da lugar a quimeras responsables del síndrome CAH-X (SCAH-X). Los pacientes con este síndrome presentan manifestaciones clínicas de hiperplasia suprarrenal congénita (HSC) y síndrome de Ehlers-Danlos (SED). La descripción del SCAH-X es reciente y es limitado el número de estudios disponibles. El objetivo de este trabajo es poner a punto un abordaje para la detección de todos los tipos de quimeras CAH-X, determinar su frecuencia y la distribución en población española así como valorar la expresividad clínica en un grupo de pacientes.

**Métodos:**

se seleccionaron 186 pacientes candidatos al estudio molecular CAH-X. Dicho abordaje molecular incluyó la técnica MLPA, detección de heterodímeros por electroforesis en gel capilar y secuenciación de exones 40, 41 y 43 de *TNXB*. La revisión de historias clínicas y la evaluación de signos y síntomas SED se ha llevado a cabo en 20 pacientes de tres Hospitales de referencia.

**Resultados:**

Setentaiocho pacientes HSC presentaron quimeras CAH-X (41,9 %). Se detectaron 46 quimeras CH1 (24,7 %), 24 CH2 (12,9 %) y 8 CH3 (4,3 %), con una distribución geográfica no homogénea. Siete de los 20 portadores de quimera CAH-X valorados clínicamente (35 %) presentaron manifestaciones clínicas asociadas a SED.

**Conclusiones:**

La implementación del abordaje molecular descrito en este trabajo ha permitido determinar el impacto del SCAH-X en población española. La expresividad clínica detectada y la considerable prevalencia del SCAH-X hacen recomendable el diagnóstico temprano de esta entidad para realizar un adecuado seguimiento de las manifestaciones clínicas que lo caracterizan.

## Introducción

La hiperplasia suprarrenal congénita (HSC) comprende un conjunto de alteraciones endocrinas autosómico-recesivas (entidades bialélicas), que son consecuencia del déficit específico de alguna de las enzimas o proteínas transportadoras que participan en la biosíntesis suprarrenal del cortisol y/o aldosterona (OMIM #201910). El déficit de la enzima 21-OH (21-OHD), consecuencia de alteraciones en el gen *CYP21A2,* es responsable del 95 % de los casos de HSC [1], [2], [3]. Existen básicamente dos formas clínicas, dependiendo del grado de afectación de la actividad de la enzima 21-OH [1], [2], [3]: las formas neonatales clásicas (CL: pérdida salina-PS o virilizantes simples-VS), que afectan a 1:12,000–1:14,000 nacidos vivos [4, 5], son graves, presentan alteraciones graves en ambos alelos y se caracterizan por una virilización variable de genitales externos femeninos e insuficiencia suprarrenal con o sin pérdida salina. Las formas no clásicas (NC) afectan a 1:100–1:1,000 nacidos vivos [4] y, aunque pueden ser incluso asintomáticas, pueden presentar en uno de sus alelos una alteración grave.

**Figura 1:**
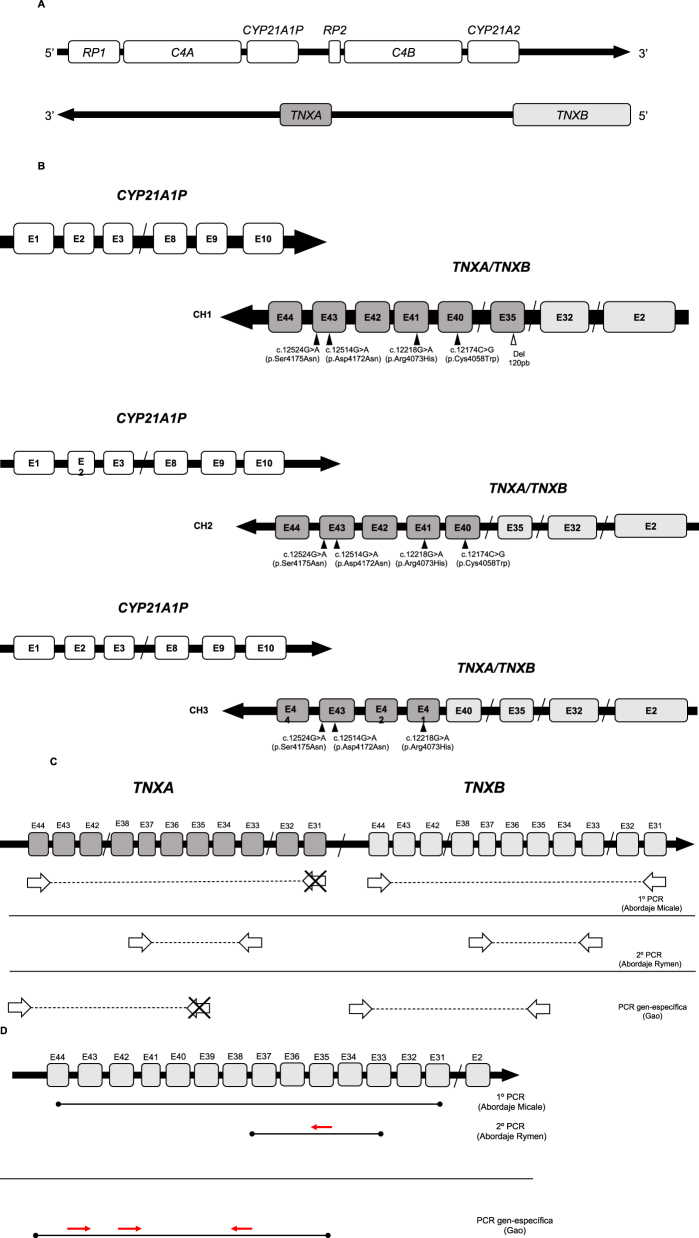
Organización genómica del locus 6p21.3, quimeras CAH-X y su caracterización molecular. (A) Locus 6p21.3 y módulo RCCX donde aparecen esquematizados los genes y los respectivos pseudogenes que lo conforman (Adaptación de Marino, 2021). (B) Representación esquemática de los 3 tipos de quimeras CAH-X (CH1, CH2 y CH3) encontradas hasta la fecha. Los cuadros representados en gris claro corresponden a los exones de *TNXB* y los representados en gris oscuro son los correspondientes a *TNXA*. En cada una de las quimeras representadas aparecen indicadas las variantes moleculares que las caracterizan (Adaptación de Marino, 2021). (C) Esquema donde aparecen representadas las distintas estrategias de amplificación de los exones de *TNXB* para el estudio de SCAH-X indicando los *primers* de amplificación empleados en las PCR junto a la publicación donde han sido propuestos (Adaptación de Marino, 2021). (D) Esquema de los amplicones obtenidos tras la realización de las PCR y los primers empleados para la secuenciación de los exones 35, 40 41 y 43 de *TNXB*.

*CYP21A2* es un gen localizado en la región III del sistema HLA (6p21.3, *human leukocyte antigen*) [6]. Próximo a él, a una distancia de 30 kb [7], se encuentra *CYP21A1P*, su pseudogén no funcional [8, 9]. Ambos comparten una homología del 98 % en exones y del 96 % en intrones [10]*.* La región se caracteriza por una particular organización estructural que incluye varias parejas de genes/pseudogenes dispuestas en tándem, convirtiéndola en una región compleja de difícil estudio [11]. Estas parejas incluyen a *RP1*, *C4A* y *TNXB* y sus respectivos pseudogenes; *RP2, C4B* y *TNXA*, los cuales tampoco son funcionales [11, 12] (Figura 1A). *TNXB* y *TNXA* están codificados en la hebra complementaria a *CYP21A2* y *CYP21A1P* y están parcialmente solapados con estos en su región 3′-UTR [13]. Mientras que *TNXB es* un gen funcional de 44 exones, *TNXA* está truncado (4,5 kb) y presenta homología con *TNXB* entre los exones 32–44 [13].

La tenascina (TNX), codificada por *TNXB*, es una proteína de la matriz extracelular perteneciente a la familia de las tenascinas. Es una glicoproteína expresada activamente en el tejido conectivo [14, 15] donde juega un papel estructural soportando los complejos macromoleculares de la matriz, las fibrillas de colágeno y de elastina y dotando al tejido conectivo de las propiedades biomecánicas adecuadas.

Las alteraciones monoalélicas (patrón dominante) de *TNXB* se asocian con el síndrome de Ehlers-Danlos tipo hipermóvil (SEDh) (*International EDS Consortium)* [16]. El SED es un grupo heterogéneo de trastornos hereditarios del tejido conectivo caracterizados por presentar hipermovilidad articular, hiperextensibilidad y fragilidad de la piel [17]. Estos síndromes presentan una prevalencia de 1:5,000 en población general [18] y su causa se debe a un espectro de alteraciones genéticas que implican frecuentemente al colágeno y su metabolismo [19].

La disposición en tándem y a la alta homología entre *CYP21A2* con *CYP21A1P* y *TNXB* con *TNXA* facilitan el apareamiento asimétrico que puede dar lugar a fenómenos de conversión y reordenamiento que suponen el principal origen de las alteraciones moleculares detectadas en esta región. En función de dónde tenga lugar el punto de ruptura, se pueden generar diferentes híbridos o quimeras, también denominados “deleción 30 kb”: 1) Si la recombinación asimétrica tiene un punto de ruptura intragénico en *CYP21A2*, solo se verá afectado este gen funcional [12]. 2) Si la recombinación asimétrica presenta un punto de ruptura post-génico, los híbridos de deleción generados son susceptibles de involucrar también a *TNXB/TNXA* [12]. En este caso, puede aparecer una sintomatología clínica adicional derivada de la afectación funcional de TNX, reconocida hoy como síndrome CAH-X (SCAH-X) [19], [20], [21].

Se han descrito tres quimeras CAH-X (CH1, CH2, CH3) según la localización del punto de ruptura (Figura 1B). La quimera CH1 incluye la deleción de 120 pb (Del120pb) y da, por ello, lugar a una haploinsuficiencia [22]. La quimera CH2 se caracteriza por la alteración (NM_019105.8) c.12174C>G (p.Cys4058Trp) del exón 40 [20]. La quimera CH3 presenta el *cluster*: (NM_019105.8) c.12218G>A (p.Arg4073His), exón 41; c.12514G>A (p.Asp4172Asn) y c.12524G>A (p.Ser4175Asn), exón 43 [13]. Las proteínas codificadas por las quimeras CAH-X CH2 y CH3 presentan cambios estructurales que serían peor tolerados al ejercer un efecto dominante negativo que daría lugar a una mayor gravedad en la expresión clínica [20, 23, 24].

Los manifestaciones clínicas del SCAH-X son anomalías del tejido conectivo: alteraciones músculo-esqueléticas (hipermovilidad articular generalizada, subluxaciones, artralgias crónicas), dermatológicas (hiperextensibilidad de la piel, cicatrización anómala), cardíacas (defectos congénitos, dilatación aurículo-ventricular) y/o alteraciones digestivas (hernias, prolapsos) [25, 26]. Las series más relevantes recientemente publicadas [13, 19, 22, 24, 27] definen la prevalencia del SCAH-X en diferentes poblaciones y analizan las manifestaciones clínicas. En series en las que predominan las formas CL, la prevalencia se acerca al 10–15 % [13, 19, 22, 24, 27]. El diagnóstico de SCAH-X es clínico, está basado en la existencia de alguna de las manifestaciones clínicas mencionadas en pacientes HSC. Actualmente, no se dispone de un tratamiento específico para este síndrome, pero su evolución clínica mejora realizando revisiones periódicas de seguimiento y planteando medidas de soporte y rehabilitación [22].

El objetivo de este trabajo es conocer en población española la frecuencia del SCAH-X en pacientes con HSC y la distribución de frecuencias de los distintos tipos de quimeras con el propósito de promover el examen clínico de estos pacientes que pudiera contribuir a un adecuado seguimiento e intervención clínica en caso de que fuera necesario.

## Materiales y métodos

Se trata de un estudio retrospectivo, observacional y descriptivo. No obstante, el rápido avance del conocimiento a nivel internacional del SCAH-X [15] y su relevancia, han hecho necesario incorporarlo de manera prospectiva en la dinámica asistencial. La población inicial de estudio (n=4,157 casos índice) la forman los pacientes con sospecha diagnóstica de HSC provenientes de todo el ámbito nacional cuyo análisis molecular de *CYP21A2* se realizó en el Laboratorio de Diagnóstico Molecular del HGUGM (2000–2022) y en el laboratorio del Hospital Universitario La Paz (1995–2000). En base a los datos moleculares de los pacientes HSC ya genotipados, se seleccionaron los pacientes a riesgo de padecer SCAH-X por presentar, al menos, un alelo con deleción-conversión del gen *CYP21A2* susceptible de involucrar también a *TNXB.* La serie incluye todo el espectro de formas clínicas asociadas al 21OHD*:* CL (PS, VS), NC, crípticas y portadores con hiperandrogenismo funcional (HF), ya que en todas ellas podemos encontrar al menos un alelo con deleción *CYP21A2*. De los 4,157 casos índice, 370 (8,9 %) presentaban, al menos, un alelo con deleción-conversión de *CYP21A2*. Ciento ochenta y cuatro (49,7 %) presentaban un punto de ruptura intragénico que no implicaba a *TNXB*, por lo que no fueron candidatos al estudio. Los 186 restantes (50,3 %) presentaban un punto de ruptura más allá del exón 6 de *CYP21A2* (post-génico) y, eran potencialmente susceptibles de incluir alteraciones en *TNXB*. Se analizaron también 32 familiares afectos y 1 individuo (paciente con HSC) al que el estudio SCAH-X le fue solicitado expresamente. En total se analizaron 186 familias. En 9 familias no se disponía de DNA del caso índice pero en 6 de ellas se pudo analizar DNA de familiares no afectos que segregaban el alelo a investigar. Se excluyeron del estudio los pacientes HSC que presentasen otros déficits. El estudio propuesto obtuvo el dictamen favorable del CEIm del HGUGM. Este estudio se llevó a cabo de conformidad con las premisas de la Declaración de Helsinki de 1964 y sus posteriores modificaciones.

El estudio molecular *CYP21A2* incluye técnicas como la hibridación específica de alelo (*allele-specific oligonucleotide*, ASO), secuenciación Sanger, SNaPshot y dosis génica MLPA, tal y como describe Ezquieta [28–33] revisado en Arriba y Ezquieta [34].

El análisis molecular de *TNXB* incluyó diferentes abordajes moleculares, que fueron implementados para este estudio (Tabla complementaria 1). La detección de quimeras CAH-X CH1 se realizó mediante dos abordajes: 1) Análisis de dosis génica mediante técnica MLPA, utilizando el kit empleado en el estudio de las muestras asistenciales de HSC (SALSA MLPA Probemix P050 CAH versión C1 o superiores, MRC Holland, Ámsterdam, Países Bajos) siguiendo el protocolo del fabricante que verificamos frente a la técnica de referencia original (Southern TaqI y BglII sonda pC21/3c). El análisis se realizó con el programa Coffalyzer (Coffalyser.Net™, MRC Holland, Ámsterdam, Países Bajos); 2) Cribado mediante electroforesis en gel capilar que informa de la presencia/ausencia de heterodúplex en el amplicón *TNXB* ex33s-ex37(i)as (Tabla complementaria 1) sugestivos de la existencia de Del120pb característica de la quimera CH1. Como técnica de confirmación, se secuenció el exón 35 obtenido tras la PCR anidada con el *primer* 35s (5′-TCATCGCCTCGCATTTCCTCTC-3′) diseñado en el laboratorio y se realizó MLPA. Para la detección de quimeras CH2 y CH3 se optimizó una amplificación PCR TNXB gen-específica (Tabla complementaria 1) secuenciando los exones 40, 41 y 43 de *TNXB* con dideoxinucleótidos fluorescentes (BigDye^TM^ Terminator v3.1 Cycle Sequencing Kit, Applied Biosystems by Thermo-Fisher Scientific^®^, Waltham, MA, USA). Los *primers* utilizados para la PCR gen específica y para la secuenciación de los exones de *TNXB* son los publicados por Gao et al. [22] a excepción del correspondiente para la secuenciación del exón 40, para el cual se empleó el *primer TNXB*38F propuesto por Rymen et al. [35]. El esquema con las estrategias de amplificación para las quimeras CAH-X y los *primers* de secuenciación aparecen representados en las Figuras 1C y 1D.

Todas las PCR mencionadas se realizaron en un termociclador ProFlex^TM^ PCR System (Applied Biosystem^®^, Waltham, MA, USA). La purificación de los amplicones obtenidos se realizó con el kit QIAquick^®^ PCR Purification Kit (QIAGEN GmbH, Hilden, Alemania). La secuenciación de los mismos, así como el procesamiento de las muestras de la técnica MLPA, se realizaron en un analizador genético ABI PRISM 3730xl (Applied Biosystem^®^, Waltham, MA, USA) de la Unidad de Genómica del HGUGM. Los programas informáticos empleados para el análisis de todas las secuencias obtenidas fueron: Chromas y Sequence Scanner Software 2.0 (Applied Biosystem^®^, Waltham, MA, USA).

Los resultados de los pacientes con quimeras SCAH-X correspondientes a los hospitales universitarios terciarios La Paz, Virgen de la Arrixaca y Marqués de Valdecilla fueron informados a los clínicos responsables que adoptaron, en cuanto al tipo de evaluación clínica a realizar, la decisión más viable (reevaluación de la historia clínica en todos los casos, consulta presencial si resultó posible o telefónica y/o realización de pruebas complementarias, algunas de ellas por el momento solo programadas). Nótese que el desarrollo del estudio coincidió con la “etapa COVID” que limitaba la presencia de pacientes y familiares a los centros hospitalarios. Las manifestaciones clínicas detectadas fueron recogidas en una tabla diseñada para tal fin en una hoja Excel basada en la descrita por Miller y Merke [25].

Se realizó un análisis estadístico descriptivo. Para las variables cuantitativas y cualitativas se calcularon las frecuencias y los porcentajes. La comparación de nuestros resultados con los publicados en otros trabajos se realizó mediante un contraste de hipótesis. Se consideraron diferencias estadísticamente significativas cuando p<0,05.

## Resultados

### Frecuencia y distribución de la quimeras CAH-X en población española

**Figura 2:**
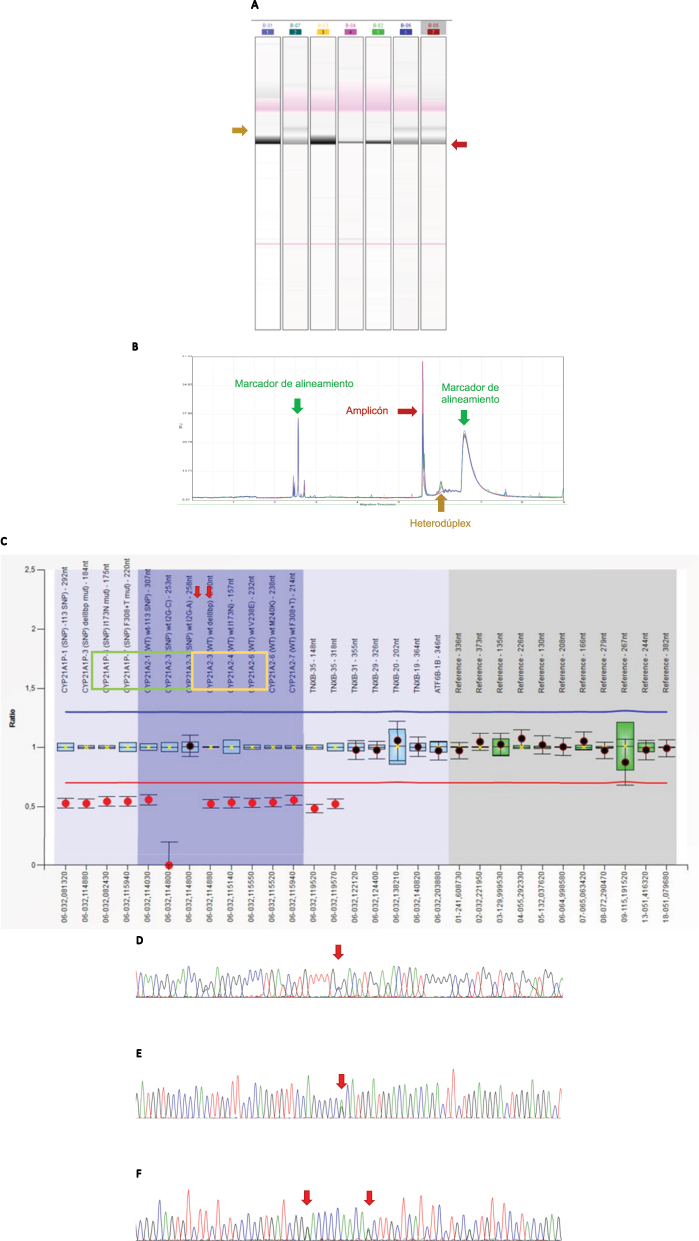
Estrategia molecular para quimeras CAH-X. (A–B) Electroforesis en gel capilar. (A) Ejemplo de una carrera donde aparecen varias muestras positivas (carriles 2, 6 y 7) las cuales presentan la banda correspondiente al heterodúplex (indicado en color mostaza). El resto de muestras son negativas para la Del120pb. (B) Perfil del cromatograma obtenido cuando una muestra es positiva para el cribado de heterodúplex. Se encuentran indicados los picos correspondientes a los marcadores de alineamiento (color verde), al amplicón (color granate) y al heterodúplex formado (color mostaza). (C) Resultados obtenidos por MLPA: visualización de los resultados correspondientes a un paciente con Del120pb (exón 35 *TNXB*). En verde: sondas correspondientes al gen *CYP21A2*. En amarillo: sondas correspondientes a *TNXB*. Flechas rojas: indican las sondas que hibridan contra el exón 35 del gen *TNXB*. (D–F) Secuencias correspondientes a las regiones donde se encuentran las variantes a estudio características del SCAH-X. (D) Exón 40 de *TNXB*, donde aparece señalada con una flecha la posición c.12174C>G (p.Cys4058Trp), correspondiente a un paciente heterocigoto para la variante analizada. (E) Exón 41 del gen *TNXB*. La flecha indica la posición c.12218G>A (p.Arg4073His), característica de la quimera CAH-X CH3. (F) Región correspondiente al exón 43 del gen *TNXB*. Las flechas indican, respectivamente las posiciones c.12514G>A (p.Asp4172Asn) y c.12524G>A (p.Ser4175Asn) (ambas heterocigotas para las variantes analizadas).

El abordaje optimizado para la detección de quimeras CAH-X CH1, CH2 y CH3 se presenta en la Tabla Suplementaria 1 y las características de la serie analizada molecularmente (186 pacientes, ver Materiales y métodos) en la Tabla 1. En la Figura 2 se muestran ejemplos de los resultados obtenidos con las diversas técnicas.

**Tabla 1:** Características de los pacientes analizados en la serie.

	Hombres (n=65)	Mujeres (n=121)
Edad media, años	25,7 (0,6–55,3)	23,8 (2,5–58,9)
Formas HSC, n		
PS	41	33
VS	5	12
NC	17	65
HF	2	11

HF, hiperandrogenismo funcional; HSC, hiperplasia suprarrenal congénita; NC, formas no clásicas de HSC; PS, forma clásica pérdida salina; VS, forma clásica virilizante simple.

En la Tabla 2 se muestran los resultados de este estudio junto al resto de series analizadas hasta el momento. Setenta y ocho pacientes presentaron alguna de las quimeras CAH-X (41,9 %). En lo que se refiere a la quimera CAH-X CH1, el estudio de 73 muestras analizadas asistencialmente con MLPA que incluía sondas TNXB (Materiales y métodos) permitió la detección de 30 pacientes CH1. El análisis retrospectivo mediante el cribado de heterodúplex en 84 pacientes permitió la detección de 16 individuos adicionales, confirmados mediante MLPA. En total, 46 pacientes presentaron la quimera CH-1 (24,7 %, 46/186), 24 presentaron la quimera CH-2 (12,9 %, 24/186) y ocho la quimera CH-3 (4,3 %, 8/186). Se confirmó la segregación familiar en 21 familiares no afectos HSC pero sí portadores de alelos CAH-X.

**Tabla 2:** Datos obtenidos en los estudios de prevalencia de SCAH-X en las diferentes cohortes.

Estudio	Lao, Merke [27]	Gao [22]	Marino [13]	Paragliola [19]
Pacientes HSC analizados^a^, n	135	424	337	196
Formas clínicas, n	N.D.	PS 137	N.D.	PS 99
		VS 249		VS 42
		NC 38		NC 55
Pacientes con DelB^b^, n (%)	72 (29)	94 (22)	66 (19,5)	74 (37,8)
Pacientes con SCAH-X^c^, n	21	59	48	21
Prevalencia SCAH-X sobre el total de pacientes analizados, %	15,6	13,9	14,2	10,7
Prevalencia SCAH-X sobre los pacientes DelB, %	29	62,8	72,7	28,4
CAH-X CH1, n (%)	13 (18,1)	35 (59)	26 (54,2)	13 (61,9)
CAH-X CH2, n (%)	8 (11,1)	13 (22)	21 (43,8)	7 (33,3)
CAH-X CH3, n (%)	0	11 (18,6)	1 (0,9)	1 (4,8)

^a^Número de casos índice. ^b^Deleción situada en *CYP21A2,* teniendo en cuenta diferentes puntos de ruptura (intragénico y postgénico). ^c^Incluye pacientes monoalélicos y bialélicos. DelB, deleción-conversión en *CYP21A2*; HSC, hiperplasia suprarrenal congénita; NC, formas no clásicas de HSC; N.D., no disponible; PS, forma clásica pérdida salina; SCAH-X, Síndrome CAH-X; VS, forma clásica virilizante simple.

En nuestra cohorte, encontramos 2 pacientes homocigotos para CAH-X, ambos formas CL de la deficiencia; uno CAH-X CH1 (CH1/CH1) y otro para CAH-X CH2 (CH2/CH2).

**Figura 3:**
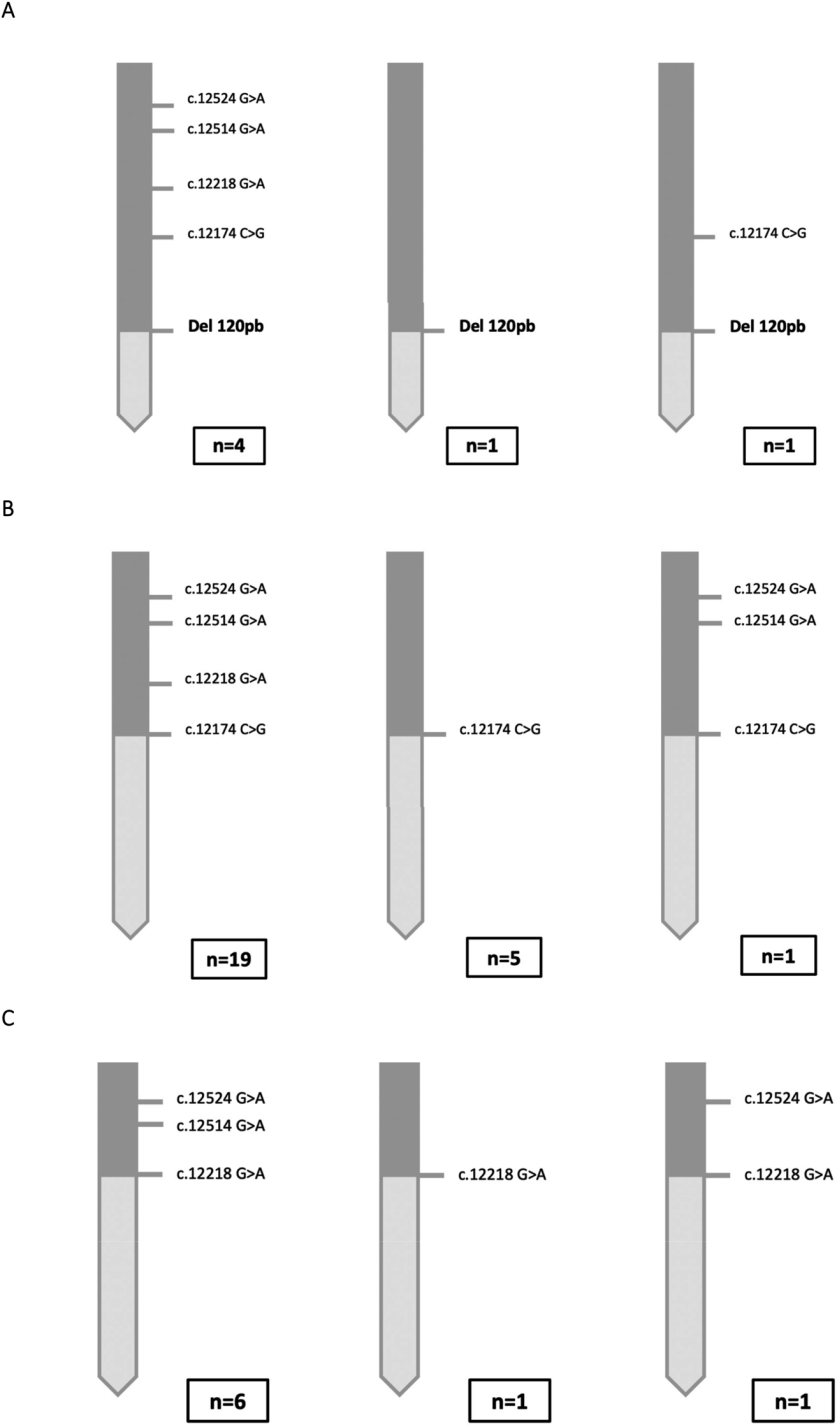
Haplotipos con las diferentes variantes de las quimeras CAH-X encontradas en los pacientes analizados. (A) Variantes de la quimera CAH-X CH1. El alelo más frecuente fue el que incluye la presencia de la Del120pb junto con las otras cuatro variantes. Se encontró un alelo que solo incluía la Del120pb y otro que, además de la Del120pb, también incluía la variante c.12174C>G (p.Cys4058Trp). En el caso de la quimera CAH-X CH1 solo se realizó el análisis molecular completo de 6 alelos. Esto es así debido a que, en el caso de estas quimeras, una vez detectada la presencia de la Del120pb mediante el cribado por electroforesis de gel capilar o técnica MLPA, no se continuó el estudio molecular de los exones 40, 41 y 43. (B) La variante de la quimera CAH-X CH2 más frecuente fue la que incluía la alteración c.12174C>G (p.Cys4058Trp), junto con las alteraciones moleculares presentes en los exones 41 y 43. La segunda variante más frecuentemente encontrada fue la que únicamente incluía la alteración característica de esta quimera. (C) Al igual que ocurre con las otras dos quimeras, la variante más frecuente de la quimera CAH-X CH3 fue la que incluía el *cluster* que la caracteriza. Se encontraron dos tipos de variantes más; una que solo presentaba la alteración del exón 41 c.12218G>A (p.Arg4073His) y otra que presentaba la alteración del exón 41 y una de las alteraciones puntuales del exón 43 c.12524G>A (p.Ser4175Asn).

Se han detectado diversas variantes de las quimeras CAH-X (Figura 3). En todas ellas, el tipo más frecuente fue el que incluye todas las variantes de *TNXA* hasta la posición del punto de ruptura.

### Análisis comparativo de frecuencias con otras poblaciones

Una comparación de frecuencias de quimeras CAH-X en pacientes HSC requiere que las series de pacientes comparadas sean homogéneas en cuanto a las formas clínicas incluidas, ya que es distinta la frecuencia de alelos portadores de deleciones/conversiones en las distintas formas clínicas: 25 % en las formas CL [28], 5 % en las formas NC [36] y 1 % los portadores con HF [36] en nuestra población. Una comparación más real puede obtenerse si se refiere exclusivamente a los pacientes HSC con alelos quimera (sea cual sea su forma clínica). En ese caso vemos (Tabla 2) que la frecuencia de alelos CAH-X en nuestra población (21,1 %; 78/370) no es distinta a las series de Lao y Merke 2019 (29 %; 21/72) [27] y Concolino 2022 (28,4 %; 21/74) [19]. Las series de Gao [22] y Marino [13], por el contrario, muestran frecuencias considerablemente más altas 62,8 % (59/94) y 72,7 % (48/66) (p<00,001), respectivamente, que podrían ser atribuibles a una constitución heterogénea de las series en cuanto el punto de ruptura de las quimeras. Excluyendo en nuestra serie las quimeras con punto de ruptura intragénico previo al exón 6 (que no podría involucrar a *TNXB*) la frecuencia de quimeras CAH-X es del 41 %. No podemos calcular este dato en las otras series porque la definición molecular de la casuística a este nivel no se precisa.

No se detectaron diferencias en la distribución de frecuencias de los distintos tipos de quimeras. La quimera CAH-X CH1 fue la más frecuente en todas las series, mientras que la CH3 fue la que se encontró en un menor número de individuos.

### Expresividad clínica

Se ha podido realizar, hasta el momento, la evaluación clínica de 20 casos índice procedentes de los hospitales mencionados (Hospital Universitario La Paz, Hospital Clínico Universitario Virgen de la Arrixaca y Hospital Universitario Marqués de Valdecilla). De estos, cinco presentaban la quimera CH1, cinco la quimera CH2 y 10 no presentaban ninguna de las alteraciones de TNXB analizadas. De los pacientes que presentaban algún alelo SCAH-X se identificaron manifestaciones clínicas en siete de ellos; 4/5 pacientes con la quimera CH1 y 3/5 portadores de CH2 (Tabla 3).

**Tabla 3:** Hallazgos clínicos identificados en los pacientes HSC con quimeras CAH-X a los que se les ha realizado la evaluación clínica procedentes de los hospitales indicados (Hospital Universitario La Paz Hospital Universitario Marqués de Valdecilla y Hospital Virgen de la Arrixaca).

Paciente	Sexo	Fenotipo HSC	Manifestaciones músculo esqueléticas	M. dermatológicas	M. Gastrointestinales	M. cardíacas	Otras
**CAH-X CH1**							

P-004	F	PS		Hematomas			
P-053	M	NC					
P-098	F	NC	Luxaciones	Hiperextensibilidad de la piel			
P-134	M	HF	Luxaciones, artralgia crónica				
P-154	F	NC		Cicatriz hipertrófica tras laparoscopia (colecistectomía), hematomas en extremidades inferiores		Evaluación cardiológica normal	Pies planos en la infancia

**CAH-X CH2**							

P-032	F	HF					Enfermedad Perthes, Bulla gigante pulmón
P-041	M	PS	Artralgia crónica, luxaciones, tendinitis/bursitis/fascitis		Colitis/proctitis		
P-062	M	PS	Tendinitis/bursitis/fascitis				
P-074	F	NC					
P-144	F	VS	Artralgia crónica (dedos manos, nudillos, muñecas y algo en cuello), escoliosis leve, tendinitis tendones extensores de primer dedo	Facilidad para hematomas		Evaluación cardiológica normal	

F, sexo femenino; HF, hiperandrogenismo funcional; HSC, hiperplasia suprarrenal congénita; M, sexo masculino; NC, formas no clásicas de HSC; PS, forma clásica pérdida salina; SCAH-X, Síndrome CAH-X; VS, forma clásica virilizante simple.

## Discusión

La descripción del nuevo síndrome SCAH-X que afecta de una forma evolutiva a los pacientes HSC supone un reto para los facultativos implicados en el seguimiento de esta frecuente entidad congénita. Un óptimo seguimiento de los pacientes debería incluir la evaluación de los signos clínicos que lo caracterizan [15] que no son los habitualmente contemplados en la HSC. Una orientación más precisa, definiendo los pacientes que presentan alelos quimera CAH-X (que implican a ambos genes, *CYP21A2* y *TNXB)* lo facilitaría. La estrategia molecular descrita en este trabajo permite la detección e identificación de las variantes en *TNXB* características de las quimeras CAH-X. La técnica MLPA que se utiliza para el estudio de dosis génica *CYP21A2* en el diagnóstico molecular HSC solo en sus versiones más recientes ha permitido la búsqueda sistemática de quimeras CAH-X, aunque solo permite la detección de la quimera CAH-X CH1 que, si bien es la mayoritaria, podría no ser la que asocia las formas más graves SCAH-X [15]. Resulta imprescindible un abordaje complementario, como el que aquí se describe, para las quimeras CH2 y CH3. Por otro lado, el estudio retrospectivo, necesario porque las técnicas anteriormente utilizadas no permitían detectar estas quimeras, puede beneficiarse del cribado de heterodímeros propuesto que detecta CH1.

Este estudio es el más amplio hasta el momento y ha abarcado la totalidad de pacientes con sospecha HSC analizados. Se han incluido no solo las formas clínicas graves, PS y VS, sino también, las muy numerosas formas que también pueden presentar un alelo grave portador de deleción/conversión, NC, crípticas e incluso las formas monoalélicas (portadores con HF). Con el fin de optimizar el rendimiento diagnóstico se ha realizado una selección molecular dirigiendo el estudio a los pacientes HSC que presentaban los alelos *CYP21A2* susceptibles de implicar a *TNXB*, es decir aquellos que presentan quimeras con punto de ruptura en la región 3′ y conversiones grandes que se extienden más allá de *CYP21A2*.

En los estudios previos de prevalencia SCAH-X el espectro clínico analizado ha sido más limitado: los iniciales solo analizaban formas CL [23], en algunos [13, 27] ni siquiera se definía este aspecto, y solo en los últimos estudios se incluyen y definen las formas clínicas incluyendo algunas formas leves de la deficiencia [19, 22]. Ni siquiera la definición del concepto de “deleción de 30-kb” en los distintos trabajos ha sido comparable como señala Concolino [15]. Todo ello ha llevado a que las frecuencias CAH-X obtenidas en los distintos estudios sean distintas (Tabla 2).

En un examen rápido, podría resultar llamativa la baja prevalencia del SCAH-X detectada 1,9 % (Tabla 2). Sin embargo, un análisis centrado en datos comparables, dirigido a los pacientes HSC que presentan deleciones/conversiones (Tabla 2) nos muestra frecuencias similares con respecto algunos de los estudios: 21,1 %, 29 % y 28,4 % con los estudios de Lao, Merke y Concolino [19, 27], mientras que las de otros, Gao [22] y Marino [13], resultan considerablemente más altas. Esta elevada frecuencia podría ser atribuible a una mayor representación de quimeras *CYP21A2* con punto de ruptura en 3′ en estas últimas series. La representación diferencial de alelos en unas u otras áreas geográficas por diseminación preferente se ha descrito en HSC y se atribuye a efecto fundador deriva genética, ventaja de portadores entre otros [37–40].

En lo que se refiere a la expresividad clínica, el estudio preliminar se ha realizado en tres centros terciarios geográficamente dispersos (Cantabria, Madrid y Murcia). Hemos detectado que muchos pacientes (aunque no todos los portadores de alelos SCAH-X) presentaban manifestaciones clínicas relacionadas con SED, siendo las más frecuentes las músculo-esqueléticas. En nuestra cohorte, es por el momento limitada la disponibilidad de datos clínicos tanto en número como en profundidad de la evaluación (pruebas complementarias no realizadas, limitada a historia clínica sin evaluación dirigida a SED). Ninguno de los dos pacientes homocigotos para SCAH-X detectados ha podido ser evaluado clínicamente. La posible relación genotipo/fenotipo entre la gravedad y el tipo de quimera o la homo o heterocigosidad no ha podido todavía analizarse.

Hasta la fecha, el número de casos SCAH-X publicados en la literatura es muy reducido y no todos los pacientes analizados molecularmente han estado disponibles para llevar a cabo la evaluación clínica correspondiente (especialmente los pacientes con quimeras CH2 y CH3). A pesar de ello, resulta evidente que un seguimiento y actuación clínica cuidadosos y acordes debería mejorar el pronóstico de estos pacientes. De nuevo, la escasez de pacientes documentados y el corto tiempo de evolución que ha podido estudiarse impiden establecer conclusiones al respecto.

Este trabajo constituye el primer estudio de prevalencia del SCAH-X en la población española y ha contribuido con la puesta a punto y validación de un protocolo de análisis de todos los tipos de quimeras causantes descritos de SCAH-X apto para cualquier laboratorio con unos mínimos medios para estudio molecular básico. Se encuentra actualmente en proceso la evaluación clínica de los pacientes que portan alelos CAH-X. Las limitaciones que estamos encontrando en cuanto a la evaluación clínica de los pacientes son las siguientes: no puede realizarse en la totalidad de portadores de quimeras CAH-X detectados y, al menos inicialmente y como consecuencia de la pandemia SARS-CoV-2, solo se reevaluaron las historias clínicas. Además, los pacientes no son examinados por el mismo facultativo dado que provienen de distintos hospitales del ámbito nacional. Tampoco se ha incluido la evaluación clínica sistemática de pacientes HSC no CAH-X que, como describe Gao [22] pueden presentar hasta en un 27 % signos EDS. Debido a que se ha realizado una selección de pacientes (aquellos con quimeras CAH-X detectadas molecularmente) para hacer viable el examen clínico, puede haber subjetividad al valorar los signos SED sabiendo que presentan este tipo de quimeras. Por último, pero no menos importante, un elevado número de los pacientes aquí analizados son “todavía” pediátricos y, dado que la aparición de signos clínicos es evolutiva y, por tanto, muy dependiente de factores como la edad del paciente, estos han podido no manifestar aún clínica relacionada con SED.

A pesar de los grandes avances en los últimos años, el conocimiento de este nuevo síndrome es todavía incompleto y son necesarios más estudios que permitan definir su impacto en los pacientes HSC en las diferentes poblaciones. Es imprescindible definir con precisión si su expresividad clínica, como tal patrón monoalélico dominante, se manifiesta en portadores y en las formas leves de la deficiencia o solo en las formas CL. Dada la frecuencia de la HSC y de sus alelos portadores de quimeras que pueden involucrar a *CYP21A2* y *TNXB* resulta recomendable diagnosticar de manera temprana el SCAH-X para prevenir y abordar las posibles manifestaciones clínicas en los pacientes portadores de estos alelos.

## Supplementary Material

Supplementary Material

## Material Suplementario

Este artículo ofrece material suplementario (https://doi.org/10.1515/almed-2023-0050).
