# Supplementary material for: Caracterización molecular de la nueva entidad clínica relacionada con la hiperplasia suprarrenal congénita, síndrome CAH-X en población española
Source: Adv Lab Med. 2023 Jul 4;4(3):268–78. [Article in Spanish] doi: 10.1515/almed-2023-0050 (PMC10701483; doi:10.1515/almed-2023-0050)
Supplement: Supplementary file 1 — Supplementary Material [file j_almed-2023-0050_suppl_001.docx]

**MATERIAL SUPLEMENTARIO**

**Tabla complementaria 1.** Reactivos empleados para la realización de las diferentes PCR gen-específicas para la caracterización molecular de quimeras CAH-X y condiciones para la realización de las mismas.

|  | |  |  |
| --- | --- | --- | --- |
| Micale, 2019 - 1º PCR | V (uL) | Condiciones amplificación PCR | |
| Long-PCR *TNXB*ex31 F | 0,5 |  | Desnaturalización inicial 95°C – 3min  Desnaturalización 95°C – 30s  *Annealing* 62°C – 30s  Extensión 72°C – 7min  72°C – 7min |
| Long-PCR *TNXB*ex44 R | 0,5 |  |  |
| dNTPs (Deoxynucleoside Triphosphate Set PCR Grade, Roche Diagnostics GmbH, Mannheim, Germany) | 1 | 30 ciclos |  |
| Expand High Fidelity Buffer, with 15 mM MgCl2 10x concentrated (Roche Diagnostics Deutschland GmbH, Mannheim, Germany) | 5 |  |  |
| Agua para preparaciones inyectables (B. Braun, Melsungen, Germany) | 41 |  |  |
| Expand™ High Fidelity PCR System (Roche Diagnostics Deutschland GmbH, Mannheim, Germany) | 0,5 |  |  |
| ADN (100 ng/uL) | 2 |  |  |
| Micale, 2019 – 2º PCR | V (uL) |  | Condiciones amplificación PCR |
| *TNXB* ex33s | 1 |  | Desnaturalización inicial 95°C – 3min  Desnaturalización 95°C – 30s  *Annealing* 58°C – 20s  Extensión 72°C – 1min  72°C – 7min |
| *TNXB* ex37(i)as | 1 |  |  |
| dNTPs (Deoxynucleoside Triphosphate Set PCR Grade, Roche Diagnostics GmbH, Mannheim, Germany) | 1 | 32 ciclos |  |
| PCR Buffer with MgCl2 10x concentrated (Roche Diagnostics Deutschland GmbH, Mannheim, Germany) | 5 |  |  |
| Agua para preparaciones inyectables (B. Braun, Melsungen, Germany) | 40 |  |  |
| Taq ADN Polymerase, 5 U/μl (Roche Diagnostics Deutschland GmbH, Mannheim, Germany) | 0,5 |  |  |
| ADN (dilución 1:100 amplicón 1ºPCR) | 1,5 |  |  |
| Gao, 2020 | V (uL) |  | Condiciones amplificación PCR |
| *TNXB* ex35(Del120pb) F | 0,5 |  | Desnaturalización inicial 98°C – 3min  Desnaturalización 95°C – 30s  *Annealing* 63°C – 30s  Extensión 72°C – 3min  72°C – 10min |
| *TNXB* 3’UTR R | 0,5 |  |  |
| dNTPs (Deoxynucleoside Triphosphate Set PCR Grade, Roche Diagnostics GmbH, Mannheim, Germany) | 0,5 | 40 ciclos |  |
| Tampón HF Phusion 5X (Thermo Fisher Scientific Inc., Waltham, Massachusetts, USA) | 5 |  |  |
| Agua para preparaciones inyectables (B. Braun, Melsungen, Germany) | 22 |  |  |
| Phusion™ High-Fidelity ADN Polymerase (Thermo Fisher Scientific Inc., Waltham, Massachusetts, USA) | 0,3 |  |  |
| ADN (100 ng/uL) | 1,5 |  |  |

ADN, ácido desoxirribonucleico; as, *antisense;* dNTPS, *deoxynucleotide triphosphates*; F, *forward*; PCR, *polymerase chain reaction*; R, *reverse*; s, *sense*; Taq, polimerasa Taq; UTR, *untraslated region;* V, volume.
